# Identifying and relating biological concepts in the Catalogue of Life

**DOI:** 10.1186/2041-1480-2-7

**Published:** 2011-10-17

**Authors:** Andrew C Jones, Richard J White, Ewen R Orme

**Affiliations:** 1 Cardiff School of Computer Science & Informatics, Cardiff University, Queen's Buildings, 5 The Parade, Cardiff CF24 3AA, UK; 2 Covalent Software Ltd, 3 Hammet Street, Taunton, Somerset TA1 1RZ, UK

## Abstract

**Background:**

In this paper we describe our experience of adding globally unique identifiers to the Species 2000 and ITIS Catalogue of Life, an on-line index of organisms which is intended, ultimately, to cover all the world's known species. The scientific species names held in the Catalogue are names that already play an extensive role as terms in the organisation of information about living organisms in bioinformatics and other domains, but the effectiveness of their use is hindered by variation in individuals' opinions and understanding of these terms; indeed, in some cases more than one name will have been used to refer to the same organism. This means that it is desirable to be able to give unique labels to each of these differing concepts within the catalogue and to be able to determine which concepts are being used in other systems, in order that they can be associated with the concepts in the catalogue. Not only is this needed, but it is also necessary to know the relationships between alternative concepts that scientists might have employed, as these determine what can be inferred when data associated with related concepts is being processed. A further complication is that the catalogue itself is evolving as scientific opinion changes due to an increasing understanding of life.

**Results:**

We describe how we are using Life Science Identifiers (LSIDs) as globally unique identifiers in the Catalogue of Life, explaining how the mapping to species concepts is performed, how concepts are associated with specific editions of the catalogue, and how the Taxon Concept Schema has been adopted in order to express information about concepts and their relationships. We explore the implications of using globally unique identifiers in order to refer to abstract concepts such as species, which incorporate at least a measure of subjectivity in their definition, in contrast with the more traditional use of such identifiers to refer to more tangible entities, events, documents, observations, etc.

**Conclusions:**

A major reason for adopting identifiers such as LSIDs is to facilitate data integration. We have demonstrated the incorporation of LSIDs into the Catalogue of Life, in a manner consistent with the biodiversity informatics community's conventions for LSID use. The Catalogue of Life is therefore available as a taxonomy of organisms for use within various disciplines, including biomedical research, by software written with an awareness of these conventions.

## Introduction

As in many areas of scientific research, there is an ever-increasing need to be able to access species-related information reliably, and to be sure that various researchers are either referring to the same entity or that they know they are not. This is particularly important to the biodiversity informatics community where they are frequently using terms and scientific names which have to be understood within the context where they appear. It is not necessarily always appreciated that this issue extends beyond biodiversity informatics to other areas in which species names are used, such as bioinformatics, biomedical informatics and ecoinformatics. The use of Globally Unique Identifiers (GUIDs) can help address this problem electronically. In this paper we explain how GUIDs - and, in particular, Life Science Identifiers (LSIDs) [1] - are being used in biodiversity informatics systems. One of the most challenging problems is to manage species names effectively, due to the variability of the concepts to which they are applied, and the majority of this paper concerns the approach we have taken to solving some of these problems in recent editions of the Species 2000 Catalogue of Life system, and strategies for addressing the remaining issues. A key requirement for the Catalogue to be used to its full potential is interoperability across application domains. For example, use of species names in biomedical literature, with the associated problems of synonymy, is an important issue [2]. Indexing of biological material and data organised by species is important [3,4], not least as a means of providing users and electronic systems with alternative search terms for species, and the Catalogue of Life is a key resource in achieving this in an effective manner. We shall see that there are some inconvenient external constraints that have been imposed on our current approach, but they do not preclude the Catalogue's use for such purposes. More generally, the basic problem addressed in this paper is one that is inevitably encountered whenever there are differences of expert opinion about the categories that should be used for classifying entities, especially when opinions develop and change over time.

## Background

### The Species 2000 and ITIS Catalogue of Life project

The Catalogue of Life (CoL) [5] is seeking to build a catalogue of all known species. It uses a distributed architecture [6], which is important in order to provide suppliers of component databases with the autonomy and control they require. Users of scientific names are faced with the problem that disagreement amongst the taxonomists who publish and organise these names will lead to different scientific names being used to refer to the same organism, and to variation in the range of organisms that a given name might refer to. In order to provide a complete "synonymic index" of all the world's species, the Species 2000 programme was set up. It is creating a catalogue of known species, with their accepted names, ambiguous and unambiguous synonyms, misapplied names, vernacular names, and some other basic data, by dynamically linking available checklist databases for different higher taxa (nodes higher than species in the taxanomic hierarchy), with the ultimate aim of complete coverage of the taxonomic hierarchy and hence all known species. In partnership with the North American ITIS organisation, it has been delivering the Catalogue of Life (CoL) in two main forms: the Dynamic Checklist, updated on the Web as the component federated databases are updated, and the Annual Checklist, a snapshot of the CoL released on CD and on the Web every year. The distinction between the Annual Checklist and the Dynamic Checklist is becoming less explicit, as the main updates at present are quarterly updates to the Annual Checklist on the Web. The uniqueness of the CoL lies in its target of creating a *complete *catalogue, organised by concepts (the species), comprising a single classification assembled from peer-reviewed checklist databases. It complements other initiatives such as uBio [7], which gathers information from a variety of resources such as published literature and web documents into its Name Bank, and provides means to organise these names.

### Role of the Catalogue of Life in a semantically-linked Web

In essence the Catalogue of Life is an electronic taxonomic checklist. Researchers can use the Dynamic or Annual Checklist in order to find the status of a scientific name of their choice and - if it is not an accepted name in the catalogue - to find the corresponding accepted name. However, this checklist is also accessible via Web Services and the complete checklist can be downloaded in various forms if desired [8]. Other major providers of species information such as the Global Biodiversity Information Facility (GBIF) [9] and the Encyclopedia of Life [10] use the Catalogue of Life in order to enhance users' searches. For example, if a user searches the Encyclopedia of Life for *Drosera aldrovanda*, one of the results (s)he will obtain is for *Aldrovanda vesiculosa*, because the Catalogue of Life holds information that *Drosera aldrovanda *is a synonym of this latter scientific name:

Accepted scientific name:

*Aldrovanda vesiculosa *L.

Synonyms:

*Aldrovanda verticillata *Roxb.

*Drosera aldrovanda *F. Muell.

It can be seen that the catalogue can be used as a thesaurus to generate alternative search terms. However, there are two significant problems:

1. The combination of accepted name and synonyms can be regarded as the Catalogue of Life's definition of a single species. If in future editions of the catalogue the list of synonyms changes, this may be due to a change of opinion about the circumscription of the species and hence a change of opinion about which synonyms are applicable.

2. The *circumscription *(range of variation) associated with each of these names may be different.

**Figure 1:**
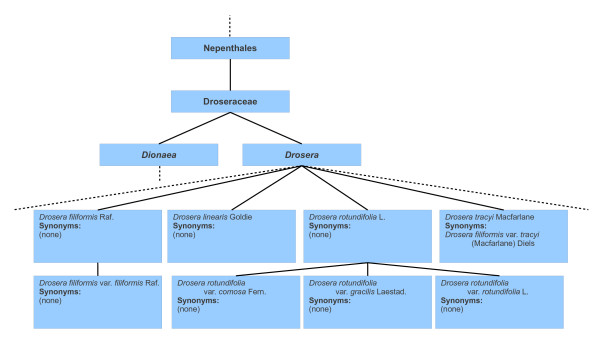
**Extract from 2008 Droseraceae classification**. This extract from the 2008 Droseraceae classification includes four of the species belonging to the genus *Drosera*. In the 2008 classification no synonyms are provided for many of the *Drosera *species. Two of the species shown - *Drosera filiformis *and *Drosera rotundifolia *- have varieties (*Drosera filiformis *var. *filiformis*, etc.). The classification includes another genus (*Dionaea*) but we have not given any of the species for that group.

**Figure 2:**
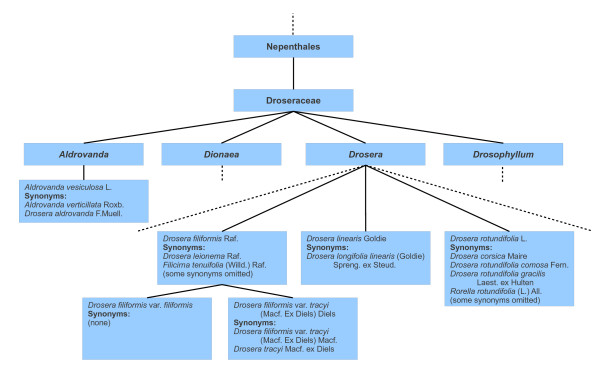
**Extract from 2009 Droseraceae classification**. This extract from the 2009 Droseraceae classification includes three of the species belonging to the genus *Drosera*. Note that one of the species in the 2008 classification, *Drosera tracyi*, does not have a corresponding species in the 2009 classification, but there is a corresponding variety (*Drosera filiformis *var. *tracyi*), as explained in the main text of this paper and illustrated in the next figure. Two additional Droseraceae genera are identified in this classification - *Aldrovanda *and *Drosophyllum *- and we discuss one of these in the text.

In order to illustrate these problems, Figure 1 shows part of the Droseraceae hierarchy as it appears in CoL 2008, and Figure 2 shows a corresponding part of the hierarchy in CoL 2009 and subsequent editions of the Catalogue. The underlying reason for the changes to the hierarchy in this particular case is that information from ITIS was used for Droseraceae up to and including 2008, while in subsequent years a specialist Droseraceae database has been used. The latter is much more complete than the former, but also reflects some differences of taxonomic opinion.

**Figure 3:**
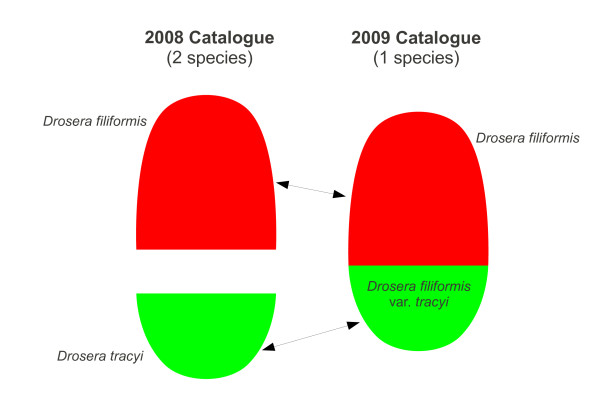
**Differing taxonomic opinion in 2008 and 2009 classification**. In the 2008 Catalogue, *Drosera filiformis *and *Drosera tracyi *were regarded as two distinct species; in the 2009 edition, they were regarded as a single species. The figure represents the circumscription (range of variation) associated with these concepts, and the mapping between the 2008 and 2009 concepts for this particular case.

Sometimes, change can occur at higher levels of the classification, but in this particular case, no such changes are evident: the family Droseraceae is placed in the order Nepenthales, and the two genera *Dionaea *and *Drosera *are included in both classifications. (The additional genera *Aldrovanda *and *Drosophyllum *in the 2009 classification appear to be omitted from the 2008 classification due to incompleteness, rather than any other reason.) However, at the species level, the species *Drosera tracyi *is present in the 2008 classification but not in the 2009 classification. Comparing the two classifications, it will be seen that in the 2009 classification *Drosera tracyi *is a synonym of the variety *Drosera filiformis *var. *tracyi*, while, in the 2008 classification, it is the opposite way around. Figure 3 illustrates the underlying difference: in 2008, *Drosera filiformis *and *Drosera tracyi *were regarded as two distinct species; in 2009, they were regarded as a single species. This illustrates the first of the above problems: the circumscription of *Drosera filiformis *in 2009 includes a concept (*Drosera filiformis *var. *tracyi*) which was treated as a separate species in 2008. Although in this case we have assumed that *Drosera tracyi *and *Drosera filiformis *var. *tracyi *have the same circumscription (represent the same concept), in general it is not necessarily the case. For example, in the 2009 classification, *Drosera rotundifolia comosa *may have a narrower circumscription than *Drosera rotundifolia *but the information available in the checklist does not tell us whether this is the case or not. This illustrates the second of the above problems.

The differences between the 2008 and 2009 Droseraceae classifications relate to the species level and below, but there is also some evidence of historical difference of opinion regarding higher levels of the classification, in particular the genus *Drosera*. As noted earlier, the species *Aldrovanda vesiculosa *has a synonym *Drosera aldrovanda*. This indicates that at least part of it has been considered to belong within the genus *Drosera *by some authors at some point.

In the above example we have illustrated how scientific names can be unstable as a means of referring to specific taxonomic concepts. It is desirable, therefore, to have a more reliable, unambiguous way of referring to a specific species concept so that it can be known whether records relating to a given species (such as maps of occurrence records, descriptions, chemical and medicinal properties, etc.) do in fact relate to precisely the same concept of the species. It is further desirable for information about the relationships between *different, but related *concepts to be known, in order that appropriate inference can be performed. For example, if species concept *A *is contained within species concept *B *then any occurrence records relating to *A *will apply also to *B*. We shall return to this point later.

### The SPICE-TIP project

At the time when the SPICE-TIP (Species 2000 Interoperability Co-ordination Environment - TDWG Infrastructure Project) project was funded (2007) the biodiversity informatics community - and particularly the Biodiversity Informatics Standards (also known as Taxonomic Databases Working Group (TDWG)) Community [11] - was starting to adopt Life Science Identifiers (LSIDs) [1] in order to label objects persistently and uniquely. However in most cases these objects were easily identifiable and immutable, such as species names, or observation events. This is similar to the originally-envisaged uses for LSIDs to (for example) label a molecular sequence [12,13]. The authors of the present paper were asked to investigate how LSIDs could be incorporated into the Catalogue of Life, which raised various issues that had not been addressed in these other projects, relating to labelling of concepts and tracking change. Another then-current development was the Taxon Concept Schema (TCS) [14] (also referred to as the Taxonomic Concept Transfer Schema), and the SPICE-TIP project was taken as an opportunity to explore the effectiveness of the TCS for representing the concepts inherent in the Catalogue of Life. The TCS had been developed on the premise that scientific names are not satisfactory as identifiers for species concepts (as we have ourselves explained above), and that a way of solving this problem is to define the concepts in relation to other concepts and their context (such as publication information) [15]. Although this does not completely solve the problem of determining which concept a scientist may have actually used (see later in the present paper), the TCS does provide a means of expressing relationships between the concepts in different taxonomies and so is suited to our purpose. Similarly, it cannot necessarily be assumed that two sources of species-related data using the same species LSID will hold information about precisely the same species, and not different concepts, but if responsible use of the LSIDs is made, then such an assumption is reasonable.

There are various alternative options for persistent unique identifiers and metadata (as we shall discuss later), but due to the context of the project the authors were constrained to the investigation of the suitability of specific technologies rather than to identify the best technologies for the task at that stage.

## Methods

As described earlier, the research reported in this paper depends on the use of Life Science Identifiers (LSIDs) and the Taxon Concept Schema (TCS). In this section we describe LSIDs and TCS in more detail; how they were introduced into the Catalogue of Life will be described later, in the Results section. We also discuss the requirements for an effective GUID system for the Catalogue of Life, which we sought to satisfy in our design and implementation, as far as was possible within the constraints we were given.

### Life Science Identifiers

The Object Management Group (OMG) defines LSIDs as:

"persistent, location-independent, resource identifiers for uniquely naming biologically significant resources" [1].

**Figure 4:**
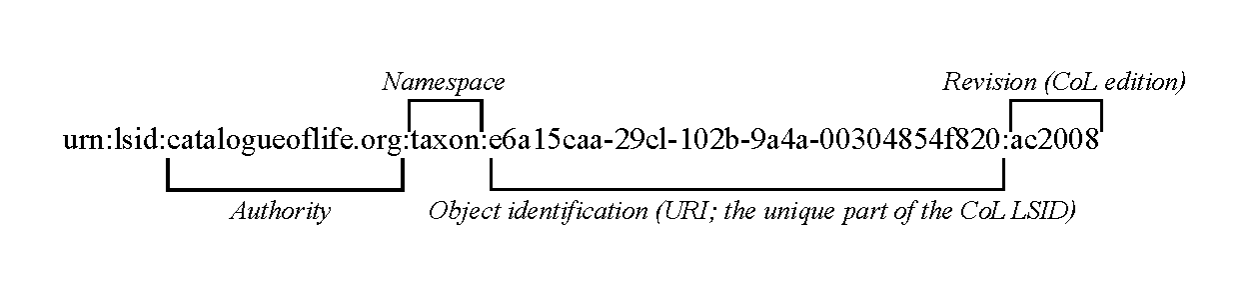
**LSID components**. A Life Science Identifier (LSID) comprises a number of components, many of which will not vary between LSIDs pertaining to the same resource. In this figure we illustrate these components using a typical Catalogue of Life LSID.

An LSID identifies a piece of byte-persistent data and/or a metadata document description. Names of taxa, among other things, are suitable candidates to be assigned LSIDs. LSIDs are Universal Resource Names (URNs) and have the constituent parts shown in Figure 4. Provided with such an identifier, a user can make use of an on-line or offline client to contact the individual authority's resolver. Page's LSID tester [16] is an example of such a client. Another facility, provided for the biodiversity informatics community, is the TDWG LSID Web Resolver [17].

The nature of the LSID resolution process (described fully in reference [12]) means that specific software must be implemented that uses the LSID resolution protocol, such as these clients. Also, to make LSIDs resolvable, the authority domain part of the LSID shown in Figure 4 requires the existence of an SRV record on the Domain Name Server (DNS). This record must refer to the actual end point (the IP address of the server and the TCP/IP port number) for the resolution service. The implementation of such a service is independent of how a client accesses it and thus, as long as it makes provisions for the standard data and metadata requests, the programming language used to develop the resolver is left up to the authority. Typical actions of a client include looking up the SRV record, calling the endpoint and parsing the RDF response when it is returned. The namespace and object identifier parts of the LSID are used by the resolver to locate or build the corresponding data or metadata from local resources such as a database. LSIDs are intended to be semantically opaque [18], so no assumptions can be made about the individual objects referred to based on the identifiers used, other than the class of the object. If this were not the case, this may lead to unwarranted assumptions and predictions being made by external clients about the internal representation of the data or dynamic formation of the authority's other LSIDs. In some cases, nevertheless, individual implementers have created a syntactical relationship between their LSIDs and the underlying data (for example, between LSIDs and a specially-constructed export schema [19]), but such relationships appear to have been adopted specifically for implementational convenience.

The end part of the LSID is the optional version field which many authorities omit altogether for the main reason that the persistent requirement of LSIDs means that change is signified by the assignment of a new identifier rather than versioning of an existing one. However, this LSID component plays an important role in the CoL implementation as we shall explain later.

### The Taxon Concept Schema

TDWG provides an ontology built on top of TCS (Taxon Concept Schema) elements to assist and standardise the RDF metadata returned by LSID resolvers [20]. Among the concepts provided by the TDWG ontology are *TaxonName *and *TaxonConcept*. This makes it possible to distinguish between the concepts which individual researchers might hold in their minds regarding a particular group of organisms which together form a taxon on the one hand, and the name(s) which might have been used for this concept on the other. The relationship between the two notions can sometimes be many-to-many. Other relation types provided include *IsParentTaxonOf *and *IsChildTaxonOf*, which make it possible to capture the hierarchical relationships within a taxonomic classification tree, and *publishedInCitation*, which makes it possible to cite supporting literature.

The TCS can be used to represent a fully organised set of taxonomic concepts, such as is the basis of the Catalogue of Life. It has also been used in other contexts, e.g. to support the process of taxonomy where the concepts and names are fluid and under debate while an attempt is being made to study and classify some group of organisms [21].

The combination of LSIDs for taxon identifiers and TCS for the information retrieved by resolving these identifiers was therefore a suitable candidate for implementing a system that uniquely labels the Catalogue of Life concepts and allows these concepts to be retrieved.

### Requirements for an effective CoL GUID system

In this section we shall discuss the requirements for an effective GUID system for the Catalogue of Life. We will later observe that the choice of GUID system (LSID) which we were asked to make meant that some of these requirements could not be met. Nevertheless, where possible we tailored our use of LSIDs with these requirements in mind.

Obvious fundamental requirements for a globally unique identifier are that it should be completely unique; that it should be permanent (even if an object is superseded - one might for example wish to access a superseded object because it was referred to in some publication), and that it should be resolvable (data or metadata about the object to which it refers must be retrievable). Others have identified such requirements and in some cases (e.g. [22]) provide a long list of further requirements, but in our view there are significant further issues that are not typically fully addressed:

1. The identifier should ideally identify a *specific *object which at any given time exists in a single, unique location, supported by a mechanism that ensures any copies of this object are up-to-date.

2. The way identifiers are used should be interoperable across disciplines. (Currently the use in biodiversity informatics is to some extent standardised, in that LSIDs are typically used and there are recommendations for the format of LSID metadata [22], but this does not automatically provide interoperability with LSID use in other domains and hence tools have to be implemented that are aware of the way these identifiers are used in the various domains.)

3. The identifier should preferably not require special tools to be installed on a user's computer in order for him or her to be able to access the objects referred to.

4. Clients should be implemented that fully support navigation via the identifiers, in a way analogous to the service provided by crossref.org (http://www.crossref.org/).

5. Where possible, a "human-friendly" option for composing LSIDs for important objects should be available, hence enabling users to have at least an initial entry point into a network of related objects. For example, in the Catalogue of Life, an identifier for each scientific name having some obvious form such as "...catalogueoflife.org:species:Drosera-filiformis" could be supported, and the object that this resolved to would contain metadata supporting navigation to the current species corresponding to this name, etc. Of course, this would be in opposition to "best practices" that have been advocated in the past [18]. An argument for identifiers not being "human-friendly" is that they are for linking data in a way that should be transparent to human users. On the other hand, it is not unreasonable for authors to want to cite globally unique identifiers in traditionally-published papers, e.g. in order to refer unambiguously to a species, and in such cases it would be convenient if the identifier could readily be typed in by the reader of such a document.

6. A means should be provided of discovering:

• GUIDs which either refer to the same object or to distinct objects that represent the same real-world entity, such as a concept

• GUIDs for related objects and concepts (including, for example, taxa which replace the current one in a checklist), and metadata about the nature of these relationships

• usage of GUIDs (electronic documents, etc, which refer to GUIDs). This last point implies that either every time a GUID is used, a reference to this usage gets recorded in a registry, or, perhaps more realistically, that an LSID crawler service of some description needs to be implemented.

The above requirements form the background to the approach taken to the implementation of Globally Unique Identifiers in the Catalogue of Life, which we shall now describe.

## Results

In order to introduce LSIDs into the Catalogue of Life, some modification of the existing Catalogue of Life software and database schemas was necessary, but in addition to this, an LSID resolver had to be set up, able to provide taxon data in response to LSID metadata requests. We shall describe these two aspects first, and then explain how relationships with concepts from other sources of taxonomic information are expressed. Finally in this section we discuss the issue of managing change - identifying when new LSIDs need to be issued.

### Modifying the CoL

SPICE-TIP (SPICE TDWG Infrastructure Project) added LSID support to both the Annual Checklist (AC) and, as an experiment, to the Dynamic Checklist (DC). As a result of this project, annual checklists from 2008 onwards contain LSIDs, and LSIDs from the 2008 AC will generally be used as examples in this paper. The CoL partners decided to use Universally Unique Identifiers (UUIDs) [23,24] as the object identifiers within CoL LSIDs, because they can be generated by a simple algorithmic process which virtually guarantees that no two UUIDs will ever be the same. This has the advantage that not only will all CoL LSIDs be globally unique (as a combination of the domain name and identifiers unique within the CoL), but the UUID is unique in its own right. It can therefore be used in contexts unrelated to LSIDs, including being used in other systems of resolvable identifiers, such as Linked Data URIs [25]. They also decided that the LSID version field should be used. Using the version field allows the reuse of an object identifier between different AC years (e.g. *:ac2008*, *:ac2009*) and between the AC and the DC (e.g. *:dc*) in cases where they refer to the same taxon. That is, if the underlying concept has not changed, the associated LSID does not change (other than the version). This allows the possibility for software or humans simply to compare the object identification parts of two LSIDs to determine whether they refer to the same taxon, and to determine which CoL edition the LSID came from, without the need to call the resolution service. The implications for taxon matching are discussed later in this paper.

In the AC, a new field was added to an existing table, containing all taxa, to hold the LSID. In the DC, the data retrieved from the contributing databases is held in a cache database. However, to incorporate LSIDs a separate database was created which would hold the data in a more suitable format than this cache. In both cases, the MySQL *UUID() *function was used to generate the object identifier LSID part. (This does not fulfil our suggested requirement for a "human-friendly" LSID composition option, but ensures the more fundamental requirement of GUID uniqueness.) In the AC, LSIDs were assigned by running the appropriate MySQL query to add them to the species (and also the higher taxa, such as genus, family, etc.) prior to being released. In the DC, code was introduced into the SPICE CAS (Common Access System) to assign LSIDs to new taxa as they enter the system. Additions were made to both the AC and DC interfaces to display LSIDs and the SPICE Web Service was modified in order to communicate them to clients including the DC interface. The modified systems also allow data providers the option to supply their own GUIDs for their species and other taxa, and also GUIDs for the names themselves (accepted names and synonyms). In contrast, within the Catalogue of Life, we have assigned LSIDs to the *concepts *it contains, not to the various names (accepted names and synonyms) which might be associated with each concept. The provider GUIDs are not made visible in the user interfaces, and are accessed via the CoL LSID resolver.

### Supporting infrastructure: resolver and metadata

The resolver for AC and DC LSIDs is written in Java using the LSID Java Toolkit available from the LSID resolution project site [26]. The resolver has access to both the AC database and the SPICE cache database in order to form the response for a metadata request. (All the CoL information associated with an LSID is held in the metadata of the object referred to by the LSID, not its data.) It also has access to what we will refer to as the "LSID Repository", which is a database that contains both a table to hold DC LSIDs and a table to hold relationships between LSIDs. The latter is consulted during every resolution process to enrich the current LSID with relationships to others (and also with relationships to other taxa in the same checklist). These may be relationships between the AC and the DC or between either the AC or DC and a provider-assigned LSID. Modification of SPICE means that this table can be populated with external LSIDs (if they are given by the provider) during the caching process.

As described earlier, the TDWG Taxon Concept Schema and TWDG ontology were used in order to represent the data that is returned by the resolver. It should be noted that the Catalogue of Life is not stored internally as a complete TCS document. A TCS document is generated in response to individual LSID resolution requests, and provides information about the taxon concerned, and about which other taxa it is related to. The most fundamental concepts used are *TaxonName *and *TaxonConcept*. As the Catalogue is a provider of a complete taxonomy using the names contained in its databases, it was decided that the core element (the one which has the *about = X *attribute where X is the current LSID) returned by the CoL's resolver for each LSID should be of type *TaxonConcept*, i.e. it is one of the agreed concepts which together comprise a consistent Catalogue of Life; each concept has a single accepted name. This could also be described as the "idea" in a person's mind when using a certain name to describe a taxon. The ontology also provides a means to relate the core *TaxonConcept *to others to better give it context by using the *hasRelationship *object property and the *Relationship *class. The *HasVernacular *and *HasSynonym *relationship types (or categories) are used to relate the current concept to its common name and synonym counterparts.

**Figure 5:**
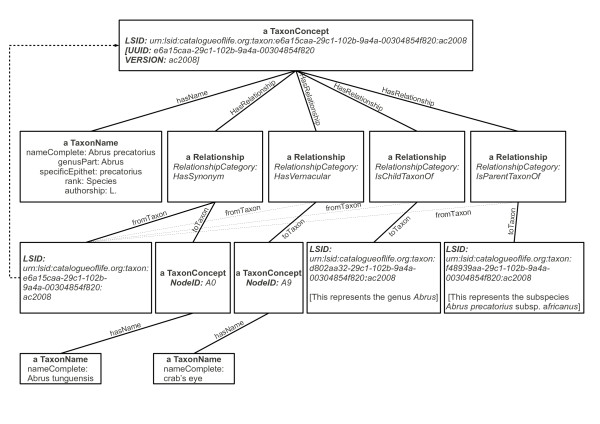
**Resolver metadata response, example 1**. This illustrates the conceptual structure of a typical response from the Resolver. It represents the species with accepted name *Abrus precatorius *L., one of the synonyms (*Abrus tunguensis*), one of the associated vernacular names (crab's eye), and a relationship to two other taxa (it is a child taxon of the genus *Abrus*, and a parent taxon of the subspecies *Abrus precatorius *subsp. *africanus*). We have represented the key concepts and their relationships; where a number of XML sub-elements have been collapsed into a single entity, their contents are given in non-italicised text; where attributes are given, the text is italicised. [See additional file 1 for a corresponding XML listing.]

In a taxonomic hierarchy it can be beneficial to view a concept in the context of its parent (the next higher taxon of which it is a member) and children (the smaller taxa which it comprises). Usefully, the TDWG ontology also has relationship types to represent hierarchical structure and these are *IsParentTaxonOf *and *IsChildTaxonOf*. Figure 5 is a conceptual illustration of a section of the metadata response for the LSID displayed in Figure 4 and shows examples of some of the previously described relationships. This LSID is for the species *Abrus precatorius *and in Figure 5 we can see its parent LSID (for the genus *Abrus*) and one of its child LSIDs (for the subspecies *Abrus precatorius *subsp. *africanus*) as well as one of its common names (of which it has several). Accepted names, common names and synonyms can all have literature references attached to them using the *publishedInCitation *although they have been removed, along with additional information, to save space in Figure 5. The ability to use LSIDs to traverse up and down the hierarchy means that all taxa are reachable via the resolver from any single LSID's metadata, i.e. the core elements of the entire catalogue can be obtained by appropriate traversal. (It will be observed that obviously this would not be a good way to perform bulk retrieval of the Catalogue.) Some clients such as the Firefox plugin can take particular advantage of this linkage, as they display additional LSIDs within a document as links so a user can manually traverse up and down the tree with simple clicks. In addition, representing the hierarchy this way opens up further possibilities for specific programmatic access.

### Relationships between CoL LSIDs and LSIDs from other providers

**Figure 6:**
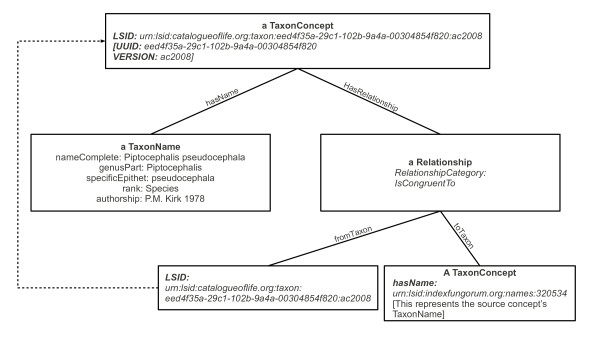
**Resolver metadata response, example 2**. This figure illustrates the conceptual structure of a typical response from the Resolver in which the classification from which a particular species has been obtained is indicated. In this case, the species *Piptocephalis pseudocephala *was provided by Index Fungorum, and the globally unique identifier used by Index Fungorum is included in the response. [See additional file 2 for a corresponding XML listing.]

We have already discussed how a given CoL LSID relates to those of its children and parent. As mentioned previously, the resolver can communicate with three different databases: extended versions of the AC and DC databases, and the LSID Repository. The latter makes provisions for relating a CoL LSID to another CoL LSID or to an externally assigned one. For example, Index Fungorum [27] is a contributor to the AC and also already provides its own LSIDs which are not the same as the CoL's, because those species from Index Fungorum included in the CoL have a distinct role as part of the CoL's classification of all known organisms. The LSIDs are nevertheless directly related. The core element returned by Index Fungorum's resolver is the *TaxonName *element from the TDWG ontology. Figure 6 illustrates an extract from a CoL LSID response for which Index Fungorum has already issued a name LSID. The TDWG ontology provides the *IsCongruentTo *relationship type for describing such links. However, a *Relationship *in the ontology is between two concepts of type *TaxonConcept *and here one subject of the relationship is a *TaxonName*. As can be seen from Figure 6, an anonymous *TaxonConcept *(not explicitly associated with any scientific names) links to the provider-assigned LSID and then this new *TaxonConcept *is used in the *IsCongruentTo *relationship.

**Figure 7:**
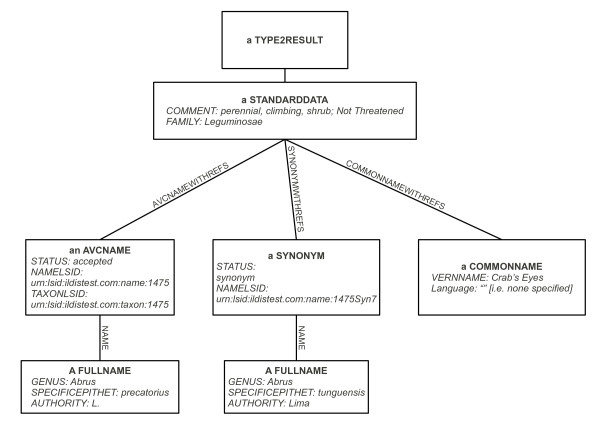
**Experimental LSID enabled wrapper response (abbreviated)**. This illustrates key elements of a typical response from our LSID-enabled wrapper implementation. [See additional file 3 for a corresponding XML listing.].

The DC communicates with its contributing databases via wrappers which convert from the databases' individual schemas (which might vary considerably) to a Common Data Model (CDM) [28]. In order to accept provider-assigned LSIDs, provisional changes were made to the CDM, which are optional on the part of the provider, to expose their own LSIDs through the use of two new attributes: *TAXONLSID *and *NAMELSID*. Figure 7 illustrates an extract from an experimental International Legume Database and Information Service (ILDIS) wrapper where these attributes have been filled with fictitious LSIDs; both attributes in the case of the accepted name but just NAMELSID in the case of the synonym. This is an example of what the CDM document defines as a "type #2" response: it contains all information about a given species (much of which has been removed from the figure, for clarity).

XML documents corresponding to Figures 5, 6 and 7 are provided [see additional files 1, 2 and 3], for readers wishing to pursue the structures described in the above subsections in more detail.

### Managing change

Most of the discussion in this paper has focussed on the assignment of LSIDs in the 2008 edition of the CoL Annual Checklist. Since the Catalogue does not only grow (by new species being introduced) but change (reflecting changing expert opinion), not all these LSIDs will be applicable to concepts in subsequent editions of the Catalogue.

Prior to the introduction of LSIDs, the CoL was criticized for using identifiers which changed from year to year [29]. The internal identifiers have never been intended to be used in other systems linking to the CoL, of course, but this criticism draws attention to the demand for persistent identifiers that are designed for use by other systems. The CoL still does not guarantee to maintain the same internal identifiers, because there appears to be no need to insist on this as a requirement, but it does now provide persistent globally unique, publicly available identifiers. The fact that some LSIDs will become deprecated through time may lead to the misunderstanding that the issue of stability still has not been addressed. It needs to be understood that in relation to *concepts *the Catalogue is intentionally not stable, so if a client is wishing to link to a name, not a concept, the client should use any LSID available for the name (or just the name itself), not a CoL-supplied taxon LSID. It should also be noted that it is intended that deprecated concepts will be accessible via their LSIDs in perpetuity, and the metadata retrieved will include information about the concepts' relationships to relevant current concepts (such as inclusion, etc.).

Due to the fact that not all suppliers of data to the CoL also provide any kind of GUID, any system to handle change must both:

• use information coming from the supplier database, where available, which indicates by a new GUID that change has occurred, and

• analyse the checklist to *detect *changes.

The first of these options is the more straightforward to implement, although it requires an understanding of each supplier's policy in order to ensure that LSIDs are changing if and only if the associated concepts are.

The second option is something that would be infeasible to undertake entirely by hand for approximately 1.5 million species. We have implemented a "Taxon Matcher" program which performs basic comparisons of species in different checklists [30]. This program is currently what is used by the CoL when issuing LSIDs for a new edition of the Catalogue. To determine whether to retain the same UUID as used for some taxon in the previous edition, or to create a new UUID, the program has to discover whether a taxon in the new edition matches one in the previous edition. Because no tracking of changing taxon concepts is currently carried out by the providers of individual checklists ("GSDs") to the CoL, it is necessary for Taxon Matcher to compare each taxon in the new edition with every taxon in the previous edition to find matching taxa.

**Figure 8:**
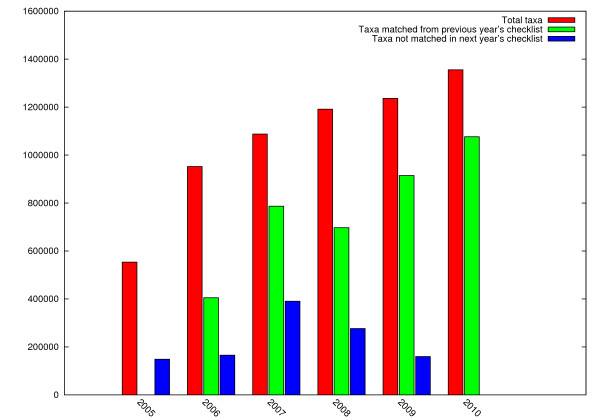
**Growth and change in the Catalogue of Life**. This graph illustrates the number of taxa (at the species and infraspecies levels only) in each Annual Checklist edition, from 2005 to 2010 inclusive. Also given is the number of taxa in each year which were also in the previous year's catalogue, and the number of taxa in each year which are not in the following year's catalogue.

Taxon Matcher is designed to accommodate a variety of requirements for documenting taxon concept change. Rather than being committed to a single view as to what constitutes a taxon concept change, it attempts to discover all changes which could be regarded as evidence of a change in taxon concept. The aim is thus to generate no "false negatives" (where a taxon has changed but the change fails to be detected), but to allow what might be "false positives", depending on an individual's point of view. It is inevitable that some entities, concepts, etc., will receive more than one identifier - this is not a problem specific to the CoL - and in future we intend to include information about known occurrences of multiple identifiers for the same concept in the metadata for the species or other taxon concerned. The UUID part of the LSID changes in every edition in which any difference is detected in several important taxon data fields. This is done by an algorithm in which a single concatenated string of data or a hashed equivalent is generated for every taxon in both editions, sorted, and then any identical pairs can be immediately located as they will be adjacent. Taxon Matcher currently compares the following data fields from each species in the Catalogue: its accepted scientific name, all synonyms, all common names, geographical distribution text, and the identifier given to it by the data provider. For a taxon above the species level, where some of the fields listed above are absent, the names of the higher taxa above it in the hierarchy are used. Users and software clients are then able to use the metadata returned by the resolver to make their own choice as to the kinds of change which they are interested in and the kinds which they wish to ignore. They can do this explicitly by processing the returned RDF themselves, or by the use of a secondary service which avoids the need to see the RDF and provides them with only the changes of which they want to be informed. Figure 8 illustrates the extent to which the Catalogue has grown and changed over a 5 year period, considering taxa at species and infraspecies (such as subspecies) level only, as estimated using our taxon matcher. It is clear that this number of concepts and rate of change cannot be handled entirely by manual means.

In future work it is intended to refine the procedure substantially, to allow editors to identify taxa with minor changes as being in fact identical, and implementing heuristics such as:

If a new synonym has been added to a taxon, and the synonym did not previously occur elsewhere in the checklist, the concept is probably unchanged.

## Discussion

In this paper we have explained how we enhanced the Catalogue of Life with Life Science Identifiers, and extended the Catalogue software to be able to respond to requests for data about individual taxa with documents in Taxon Concept Schema form, expressed as RDF. We now discuss four important issues raised by our work: the extent to which the use of LSIDs has made interoperability of the Catalogue of Life with other systems possible (especially systems for other application domains); the extent to which the precision afforded by LSIDs (or other GUIDs) might imply accuracy; the extent to which CoL LSIDs have been adopted - and the ways in which alternative GUID schemes could be supported; and the applicability of our approach to other domains.

### Interoperability

There has been a fragmentary, disjointed approach to globally unique identifiers across disciplines. Although some GUID schemes have been deployed for specific applications (e.g. DOIs [31] for publications, LSIDs for data objects in Life Science domains), and hence have had differing requirements to meet, the lack of a generally-accepted resolvable GUID specification means that the possibility of creating general-purpose software that can use GUIDs to access objects from multiple application domains is limited. One very widely used type of GUID is the UUID [24]. However, UUIDs are not in themselves resolvable, although (as we have seen in this paper) there is good reason to make them part of a resolvable identifier such as an LSID. This does in turn at least mean that we have a basis for producing more than one kind of resolvable identifier for the same object if identifier schemas that can embed UUIDs are used. This then means that such objects can be used in a wider range of application domains.

It is not the purpose of this paper to compare the various kinds of GUID that are available (Laibe & Le Novère [32] and Altman & King [33] include useful overviews in their papers), but even given the requirement that LSIDs be used, there are still decisions to be made which potentially limit interoperability:

• The TDWG community decided to use the metadata component for holding all information relating to the objects referred to by LSIDs; the data field is not used. This is in contrast with typical use of LSIDs, and means that (for example) bioinformatics software will not necessarily be able to interpret them.

• Use of TCS to describe the taxon concepts presents at least two problems. The first of these is that development of the associated TDWG ontology has been intermittent, and it is not clear whether extending some other ontology (such as the SEEK Ecoinformatics Ontology [34]) might be a more sustainable approach. The other problem is that the TCS provides its own specialised terminology for expressing taxonomic concepts and their relationship to each other. While some terminology is inevitably specialised, some (such as *IsCongruentTo *and *Overlaps*) are much more generally applicable and it would be desirable to standardise across domains so that reasoning with such relationships can be performed in a more general context.

In future work we plan to explore alternative persistent identifier schemes and to investigate how the Catalogue can be made available as Linked Data [25] in order to make the knowledge contained in the Catalogue more readily associated with other Web-accessible information.

### Precision and accuracy

Unfortunately, although the Catalogue of Life is being assembled with attention being given to the appropriate selection of species concepts for inclusion, one cannot necessarily be sure that, even if a scientist has used the Catalogue to look up a name, (s)he is using the name as the creators of the relevant part of the Catalogue had intended.

This means that although the use of LSIDs ensures that one can refer to a specific concept, present at a specific time in the Catalogue, with precision, this does not solve the problem of ensuring that scientists recording information associated with a given species concept have correctly identified the concept to use. We are currently investigating ways of detecting this problem in practice, but it seems inevitable that no complete solution will be found, because of the subjective nature of species definition. Clearly a development that would be beneficial in future is to make it easier for users to determine whether their species concepts coincide with those in the Catalogue. One current relevant development is the Encyclopedia of Life, referred to earlier, which includes descriptive species pages; another possibility would be the creation of "identification keys" linked explicitly to the Catalogue of Life classification. This would be an enormous undertaking, however, when one considers that merely enumerating the known species is a task which the Catalogue of Life has been performing for over a decade, and which it will never be able definitively to complete due to the regular discovery of new species.

### Adoption of CoL LSIDs; other GUID schemes

The adoption of CoL LSIDs has not yet been as widespread as originally anticipated, although examples of their use can be found in:

• the Belgian Species List [35], which provides CoL LSIDs in the text of its species data Web pages;

• CultureSheet dot Org, which provides CoL LSIDs for some (but not all) of the species it covers [36], and

• the Atlas of Living Australia [37], which includes CoL LSIDs in the species metadata.

There are perhaps a number of reasons for this somewhat limited adoption, which are not specific to the CoL. The end user unfamiliar with LSIDs may be disappointed to find that they cannot be resolved by simply pasting them into a web browser, although we noted earlier that browser plug-ins and web proxies exist to allow Web browsers to be used. Another reason for the relatively low rate of adoption of LSIDs may be the high profile accorded to the Semantic Web and the use of Linked Data URIs as alternative means to provide metadata about objects and concepts, especially as these are based on existing Web technology which provides less of a barrier to users unfamiliar with them.

However, because the CoL has based its identifiers on the use of UUIDs which do not necessarily require the existence of the LSID system in order to be resolved, the CoL has the option to deploy its taxon concept identifiers in other ways. For example, the CoL UUIDs can be incorporated in HTTP URIs. If this is done, the edition of the CoL from which the identifier was obtained could be omitted, and the resolution mechanism should still be able to provide metadata about which editions this taxon concept was included in and what changes, if any, occurred between them. Such changes would not relate to changes of concept, but (for example) to additional data being included. If it is important to be able to specify the edition from which the concept was obtained, then the edition code from the revision element of the LSID could simply be concatenated with the UUID part. There is no reason why the CoL cannot deploy its identifiers in multiple ways simultaneously, and so the approach we have taken allows us to accomodate changing external requirements on the provision of globally unique identifiers.

Issues relating to the choice and adoption of suitable GUID schemes are covered in detail in two recent documents commissioned by the Global Biodiversity Information Facility (GBIF) [38,39].

### Applicability to other domains

We have focussed in this paper upon the enhancement of a Catalogue of Life by adding LSIDs to the concepts defined in the Catalogue and incorporating information about relationships between these concepts in the LSID metadata. Yet concepts and variation of professional opinion are not phenomena unique to the domain of taxonomy, and not dissimilar benefits might be expected where globally unique identifiers are used in a comparable way in other domains, for example in the Unified Medical Language System (UMLS) [40], which defines the notion of a Concept Unique Identifier (CUI), and in which concepts from various biomedical taxonomies need to be related to each other.

As we have seen, some of the metadata used for taxonomic concepts is domain-specific and may not be applicable to other domains; we anticipate that the reverse would also be true. In other words, although we have been advocating the desirability of a cross-disciplinary approach to the problem of adopting GUIDs, domain-specific extensions to capture some of the nuances associated with concepts, in particular, are needed. Appropriate ontologies underpinning these extensions are also needed, if software that is not application domain-specific is to be able to make any sense of these extra details.

## Conclusions

In this paper we have demonstrated the feasibility of enhancing the Catalogue of Life with globally unique identifiers and suitable explicit metadata relating the Catalogue's concepts to each other. We have also explained how the Catalogue of Life may be regarded as a specialised ontology, providing knowledge that is needed to support semantic interoperability in biomedical and other disciplines when dealing with species-related data. Using identifiers such as LSIDs to refer to species concepts instead of using species names (which might be subject to various interpretations) can help in data integration. This is because it can be assumed (with the caveats expressed earlier in this paper) that two sources of species-related data using the same species LSID will hold information about precisely the same species, not different concepts. The provision of unique identifiers is a key aspect of the recently-commenced i4Life project [41]: i4Life aims to cross-map between taxonomies supplied by various organisations, including the NCBI taxonomy, IUCN Red List, etc., using the Catalogue of Life as a source both of names and of concepts (assemblages of names into species and other taxa) to support the cross-mapping process.

In future, there are a number of areas that would benefit from further development, including some areas in which the requirements for an effective CoL GUID system listed earlier in this paper are not yet satisfied. We have mentioned these in the previous sections but, to summarise, we are planning:

• enhanced tracking of concept changes;

• tracking LSID usage (see requirement (6));

• suitable default behaviour in cases where a CoL LSID *without *a version field is submitted to the resolver, perhaps from systems that cannot accommodate the version field. At present, the resolver fails; our intention is to change this behaviour so that the resolver will return metadata associated with the current version of the LSID.

• exploration of alternative GUID schemes and options for expressing concept metadata;

• investigation of appropriate ways to provide a "human-friendly" option for composing LSIDs for important objects (see requirement (5));

• making the Catalogue of Life available as Linked Data, and

• experimenting to determine ways of automatically identifying where users are erroneously referring to the same concept.

## Availability of supporting data

The Catalogue of Life is accessible via its Web interface at: http://www.catalogueoflife.org/

LSIDs used in the Catalogue of Life are of the form: urn:lsid:catalogueoflife.org:... and these LSIDs may be used to retrieve a TCS representation of the relevant part of the Catalogue.

The software described in this paper - enhanced Catalogue of Life (SPICE) software, software for resolving Catalogue of Life LSIDs as TCS documents, and the Taxon Matcher program - is available at: http://biodiversity.cs.cf.ac.uk/

## Competing interests

The authors declare that they have no competing interests.

## Authors' contributions

All three authors contributed to the design and approach documented in this paper. ERO implemented the software extending the Catalogue of Life to handle LSIDs and serve TCS data, with some assistance from RJW and ACJ. RJW implemented and maintains the Taxon Matcher program, and ACJ contributed some of the revisions. ACJ performed the CoL growth analysis. ACJ led the writing of the paper, with major contributions from both ERO and RJW. All authors read and approved the final manuscript.

## Supplementary Material

Additional file 1 **An abbreviated example of resolver metadata RDF response corresponding to Figure **5. This file provides the XML underlying the structure presented in Figure 5. Note that the namespace declarations have been removed; also *C: *refers to elements in the *TaxonConcept *namespace and *N: *refers to elements of the *TaxonName *namespace. The relationships between the taxon which this metadata represents (with name "Abrus precatorius") and the other taxa to which it relates are highlighted in bold face. Click here for file

Additional file 2 **An abbreviated example of resolver metadata RDF response corresponding to Figure **6. This file provides the XML underlying the structure presented in Figure 6. Note that the namespace declarations have been removed; also *C: *refers to elements in the *TaxonConcept *namespace and *N: *refers to elements of the *TaxonName *namespace. The key elements that indicate the relationship between the Catalogue of Life taxon which this metadata represents and the source taxon are highlighted in bold face (the *IsCongruentTo *relationship and the Index Fungorum LSID). Click here for file

Additional file 3 **An abbreviated experimental LSID-enabled wrapper XML response corresponding to Figure **7. The extensions to the standard Species 2000 "type #2" response which have been made in order to accommodate LSIDs are highlighted in bold face. Click here for file
