# Supplementary material for: Identifying and relating biological concepts in the Catalogue of Life
Source: J Biomed Semantics. 2011 Oct 17;2:7. doi: 10.1186/2041-1480-2-7 (PMC3245425; doi:10.1186/2041-1480-2-7)
Supplement: Additional file 1 — An abbreviated example of resolver metadata RDF response corresponding to Figure 5. This file provides the XML underlying the structure presented in Figure 5. Note that the namespace declarations have been removed; also C: refers to elements in the TaxonConcept namespace and N: refers to elements of the TaxonName namespace. The relationships between the taxon which this metadata represents (with name "Abrus precatorius") and the other taxa to which it relates are highlighted in bold face. [file 2041-1480-2-7-S1.PDF]

Additional file 1
An abbreviated example of resolver metadata RDF response corresponding to Figure 5


```
<C:TaxonConcept rdf:about=”urn:lsid:catalogueoflife.org:taxon:e6a15caa-29c1-102b-9a4a-00304854f820:ac2008”>
 <C:hasName>
  <N:TaxonName>
   <N:nameComplete>Abrus precatorius</N:nameComplete>
   <N:genusPart>Abrus</N:genusPart>
   <N:specificEpithet>precatorius</N:specificEpithet>
   <N:rank rdf:resource=”http://rs.tdwg.org/ontology/voc/TaxonRank#Species”/>
   <N:authorship>L.</N:authorship>
  </N:TaxonName>
 </C:hasName>
 <C:hasRelationship>
  <C:Relationship>
   <C:relationshipCategory rdf:resource=”http://rs.tdwg.org/ontology/voc/TaxonConcept#HasSynonym”/>
   <C:fromTaxon rdf:resource=”urn:lsid:catalogueoflife.org:taxon:e6a15caa-29c1-102b-9a4a-00304854f820:ac2008”/>
   <C:toTaxon>
    <C:TaxonConcept rdf:nodeID=”A0”>
     <C:hasName>
      <N:TaxonName>
       <N:nameComplete>Abrus tunguensis</N:nameComplete>
      </N:TaxonName>
     </C:hasName>
    </C:TaxonConcept>
   </C:toTaxon>
  </C:Relationship>
 </C:hasRelationship>
 <C:hasRelationship>
  <C:Relationship>
   <C:relationshipCategory rdf:resource=”http://rs.tdwg.org/ontology/voc/TaxonConcept#HasVernacular”/>
   <C:fromTaxon rdf:resource=”urn:lsid:catalogueoflife.org:taxon:e6a15caa-29c1-102b-9a4a-00304854f820:ac2008”/>
   <C:toTaxon>
    <C:TaxonConcept rdf:nodeID=”A9”>
     <C:hasName>
      <N:TaxonName>
       <N:nameComplete>crab’s eye</N:nameComplete>
      </N:TaxonName>
     </C:hasName>
    </C:TaxonConcept>
   </C:toTaxon>
  </C:Relationship>
 </C:hasRelationship>
 <C:hasRelationship>
  <C:Relationship>
   <C:relationshipCategory rdf:resource=”http://rs.tdwg.org/ontology/voc/TaxonConcept#IsChildTaxonOf”/>
   <C:fromTaxon rdf:resource=”urn:lsid:catalogueoflife.org:taxon:e6a15caa-29c1-102b-9a4a-00304854f820:ac2008”/>
   <C:toTaxon rdf:resource=”urn:lsid:catalogueoflife.org:taxon:d802aa32-29c1-102b-9a4a-00304854f820:ac2008”/>
  </C:Relationship>
 </C:hasRelationship>
 <C:hasRelationship>
  <C:Relationship>
   <C:relationshipCategory rdf:resource=”http://rs.tdwg.org/ontology/voc/TaxonConcept#IsParentTaxonOf”/>
   <C:fromTaxon rdf:resource=”urn:lsid:catalogueoflife.org:taxon:e6a15caa-29c1-102b-9a4a-00304854f820:ac2008”/>
   <C:toTaxon rdf:resource=”urn:lsid:catalogueoflife.org:taxon:f48939aa-29c1-102b-9a4a-00304854f820:ac2008”/>
  </C:Relationship>
 </C:hasRelationship>
</C:TaxonConcept>
```
