# Supplementary material for: Identifying and relating biological concepts in the Catalogue of Life
Source: J Biomed Semantics. 2011 Oct 17;2:7. doi: 10.1186/2041-1480-2-7 (PMC3245425; doi:10.1186/2041-1480-2-7)
Supplement: Additional file 2 — An abbreviated example of resolver metadata RDF response corresponding to Figure 6. This file provides the XML underlying the structure presented in Figure 6. Note that the namespace declarations have been removed; also C: refers to elements in the TaxonConcept namespace and N: refers to elements of the TaxonName namespace. The key elements that indicate the relationship between the Catalogue of Life taxon which this metadata represents and the source taxon are highlighted in bold face (the IsCongruentTo relationship and the Index Fungorum LSID). [file 2041-1480-2-7-S2.PDF]

Additional file 2
An abbreviated example of resolver metadata RDF response corresponding to Figure 6

<C:TaxonConcept rdf:about=”urn:lsid:catalogueoflife.org:taxon:eed4f35a-29c1-102b-9a4a-00304854f820:ac2008”>
<C:hasName>
<N:TaxonName>
<N:nameComplete>Piptocephalis pseudocephala</N:nameComplete>
<N:genusPart>Piptocephalis</N:genusPart>
<N:specificEpithet>pseudocephala</N:specificEpithet>
<N:rank rdf:resource=”http://rs.tdwg.org/ontology/voc/TaxonRank#Species”/>
<N:authorship>P.M. Kirk 1978</N:authorship>
</N:TaxonName>
</C:hasName>
<C:hasRelationship>
<C:Relationship>
<C:relationshipCategory rdf:resource=”http://rs.tdwg.org/ontology/voc/TaxonConcept#**IsCongruentTo**”/>
<C:fromTaxon rdf:resource=”urn:lsid:catalogueoflife.org:taxon:eed4f35a-29c1-102b-9a4a-00304854f820:ac2008”/>
<C:toTaxon>
<C:TaxonConcept>
<C:hasName rdf:resource=”**urn:lsid:indexfungorum.org:names:320534**”/>
</C:TaxonConcept>
</C:toTaxon>
</C:Relationship>
</C:hasRelationship>
</C:TaxonConcept>
