# Supplementary material for: Identifying and relating biological concepts in the Catalogue of Life
Source: J Biomed Semantics. 2011 Oct 17;2:7. doi: 10.1186/2041-1480-2-7 (PMC3245425; doi:10.1186/2041-1480-2-7)
Supplement: Additional file 3 — An abbreviated experimental LSID-enabled wrapper XML response corresponding to Figure 7. The extensions to the standard Species 2000 "type #2" response which have been made in order to accommodate LSIDs are highlighted in bold face. [file 2041-1480-2-7-S3.PDF]

Additional file 3

An abbreviated experimental LSID-enabled wrapper XML response corresponding to Figure 7

<TYPE2RESULT>
<STANDARDDATA COMMENT=”perennial, climbing, shrub; Not Threatened” FAMILY=”Leguminosae”>
<AVCNAMEWITHREFS>
<AVCNAME STATUS=”accepted” **NAMELSID=”urn:lsid:ildistest.com:name:1475”**
TAXONLSID=”urn:lsid:ildistest.com:taxon:1475”>
<NAME>
<FULLNAME GENUS=”Abrus” SPECIFICEPITHET=”precatorius” AUTHORITY=”L.”/>
</NAME>
</AVCNAME>
</AVCNAMEWITHREFS>
<SYNONYMWITHREFS>
<SYNONYM STATUS=”synonym” **NAMELSID=”urn:lsid:ildistest.com:name:1475Syn7”**>
<NAME>
<FULLNAME GENUS=”Abrus” SPECIFICEPITHET=”tunguensis” AUTHORITY=”Lima”/>
</NAME>
</SYNONYM>
</SYNONYMWITHREFS>
<COMMONNAMEWITHREFS>
<COMMONNAME VERNAME=” Crab’s Eyes ” LANGUAGE=””/>
</COMMONNAMEWITHREFS>
</STANDARDDATA>
</TYPE2RESULT>
